# Optical Penetration and “Fingerprinting” Analysis of Automotive Optical Liquid Silicone Components Based on Wavelet Analysis and Multiple Recognizable Performance Evaluation

**DOI:** 10.3390/polym15010086

**Published:** 2022-12-25

**Authors:** Hanjui Chang, Shuzhou Lu, Yue Sun, Guangyi Zhang, Longshi Rao

**Affiliations:** 1 Department of Mechanical Engineering, College of Engineering, Shantou University, Shantou 515063, China; 2 Intelligent Manufacturing Key Laboratory of Ministry of Education, Shantou University, Shantou 515063, China

**Keywords:** liquid silicone rubber, wavelet analysis, light transmittance, multi-recognizable performance evaluation, high-power LED molding

## Abstract

The residual stress phenomenon in the injection process of an optical lens affects the quality of optical components, and the refractive error caused by geometric errors is the most serious, followed by the degradation of the accuracy and function of optical components. It is very important to ensure that the lens geometry remains intact and the refractive index is low. Therefore, a parameter design method for an optical liquid silicon injection molding was proposed in this study. Wavelet analysis was applied to the noise reduction and feature extraction of the cavity pressure/pressure retaining curve of the injection molding machine, and multiple identifiable performance evaluation methods were used to identify and optimize the parameters of the molding process. Taking an automotive LED lens as an example, Moldex3D simulation software was used to simulate the molding of an LED lens made of LSR material, and two key injection molding factors, melt temperature and V/P switching point, were analyzed and optimized. In this paper, the transmittance and volume shrinkage of LED lenses are taken as quality indexes, and parameters are optimized by setting different V/P switching points and melt temperature schemes. The experimental results show that the residual stress is negatively correlated with transmittance, and the higher the residual stress, the lower the transmittance. Under the optimum process parameters generated by this method, the residual stress of plastic parts is significantly optimized, and the optimization rate is above 15%. In addition, when the V/P switching point is 98 and the melt temperature is 30 °C, the product quality is the best, the volume shrinkage rate is the smallest, and the size is 2.895%, which also means that the carbon emissions are the lowest.

## 1. Introduction

The development of automotive optical products has seen lens headlights become a major trend, beyond being widely used in high-grade cars, and gradually becoming popularized in low-grade models. Most traditional car mirrors use glass optical lenses, with high light transmission, high hardness, and aging resistance; these offer unparalleled advantages, but are also accompanied by high prices, ease of breakage, processing difficulties, and other shortcomings. Since then, with the development of materials, molds, and injection molding technology, PC and PMMA lenses have entered the market. Compared with glass, their good processing characteristics yield better performances, but their defects of easy yellowing also plague most automobile manufacturers. Therefore, people have begun to turn to liquid silicone.

Liquid silicone rubber (LSR) has the advantages of high light transmission (up to 95%) and excellent resistance to UV and blue light. Despite the low power and high brightness output of the new generation of LED systems, the lighting temperature of common models is up to 150 °C. The combined effects of such high temperatures and high radiation can easily accelerate the aging of existing optical thermoplastic lenses and shorten the lives of LED systems. The physical stability of silicone lenses can solve the problem of lens yellowing.

In addition, surface quality, curvature, and surface optical design are very important parameters in the development of optical lenses used to describe their optical properties. Depending on the purpose, different lenses can be used, such as simple spherical and aspherical lenses, as well as free-form and micro-structured lenses. However, not only does the geometry affect the performance and function, but the combination of materials, manufacturing processes, processing conditions, and tooling also have an impact on the optical properties.

Compared to conventional optical glass, polymer optical lenses are injection molded in a shorter time and at a lower cost, have a lighter weight and also have higher light transmission. They are used in a wide range of applications such as automotive, lighting, photovoltaic, electronics, ophthalmic and medical. For example, Fresnel lenses with micron-sized v-groove structures are used as illumination lenses for light-emitting diodes (LEDs) and are widely used in the automotive industry. Due to their micro-structured surfaces with concentric grooves, which enhance their capacity for light gathering, they are also used in photovoltaics as light collecting elements for solar cells [1]. In addition, surface quality, curvature and surface optical design are crucial to the optical performance of optical lenses, which factors affects transmittance, refraction, birefringence, reflection, internal reflection, scattering, etc. Manufacturing technologies such as injection molding IM, injection compression molding (ICM) and micro-injection molding (μIM) have been increasingly used for the production of optical components, enabling high productivity, low cost and automation, which contribute to the mass production of these components. These techniques can replicate complex geometries and microstructures and have the advantage of high reproducibility, achieving optical properties comparable to those of glass.

In 2014, Marco et al. [2] used Fresnel lenses on the microscale in a study comparing conventional injection molding (IM) with two innovative molding techniques—injection compression molding (ICM) and vacuum injection molding (VIM). They showed that ICM produced the most accurate and reproducible Fresnel lenses, while VIM achieved a minor process improvement. In 2007, Michaeli et al. found that the geometric accuracy of optical lenses could be further improved by using injection compression molding technology and a suitable mold compared to injection molding technology [3]. In the production of optical lenses, the injection compression molding technique has greater advantages. All of the above studies have shown that ICM is an optimal method for producing high-precision optical lenses compared to conventional injection molding techniques, and that high productivity, complex geometry, dimensional accuracy, and high reproducibility in high-precision optical components can be obtained using this technique.

However, for polymer optics, residual stress is an issue that deserves attention, given its serious impact on the quality of the optics. The most problematic of these is anisotropy, which affects optical properties, such as birefringence, and leads to a loss of mechanical properties. Residual stresses also lead to warpage, non-uniform refractive index distribution, and changes in radius of curvature and shape deviation. This affects the optical properties, as these parameters determine the focal length, principal plane position and wavefront aberration. Therefore, reducing the occurrence of residual stresses during the molding process can greatly help to improve the quality of optical lenses.

In 2009, Can Weng et al. [4] calculated residual stresses in microlens arrays, which are difficult to measure and characterize, using the birefringence method and predicted the distribution of residual stresses using finite element simulations. They found that the mold temperature is the most important process parameter. The higher the mold temperature, the lower the maximum residual stress. In 2021, Hanjui Chang et al. [5] proposed a method to evaluate the residual stress of products based on fuzzy theory and used the photoelastic stress compensation method for measurement verification. This method can effectively quantify and measure the residual stress of products and provide an identifiable and reliable correction basis for subsequent mold maintenance or parameter adjustment. In 2015, Macías et al. [6] found that the quality of optical components made of transparent thermoplastic polymers depends on the presence of residual stresses, and that the wrong choice of process parameters can lead to residual stresses in plastic lenses, as well as that residual stresses in the components can significantly affect the structural dimensions of the lenses and their dimensional accuracy. In 2017, Marcel Roeder et al. [7] found that three process parameters, melt temperature, mold temperature and compression force, had a significant effect on molding accuracy during the manufacturing of diffractive optical elements (DOEs) using injection compression molding. and experimentally showed that only the precise control of each step of the molding process can produce complex polymer optical elements. In addition, they pointed out that high mold temperatures and compression forces are essential to replicating microstructural features.

It can be seen that mold temperature, melt temperature and compression force play a very critical role in improving the quality of optical components. In other words, the control of the above three process parameters is an important means to improve the repeatability and consistency of product quality in the injection molding process. If they can be optimized and detected in real time and controlled with effective adjustments, then the production of optical lenses with high precision and a high reproduction rate can also be achieved.

## 2. Literature and Review

The results show that the molding quality of optical lenses is closely related to the setting of the molding process parameters. The parameter settings are optimized to achieve the goal of reducing the warpage (to reduce optical aberration) and residual stress (to reduce birefringence) caused by shrinkage displacement. However, in practice, these two performance goals are mutually exclusive. In other words, smaller warpage leads to larger residual stresses and vice versa. Lin, Chao-Ming et al. [8] conducted Taguchi experiments to determine the process parameters that minimize warpage and delay, respectively, in a symmetric plastic biconvex Fresnel lens. The gray correlation analysis technique was then applied to the Taguchi results to determine the processing parameters that achieve the best compromise between the two performance objectives. Their proposed gray-based Taguchi method provides a feasible technique for optimizing the two performance objectives of the considered biconvex Fresnel lens. and Tsai, K. M. et al. [9] also applied Taguchi’s experimental method for the first time to determine the optimal combination of parameters and significant factors affecting the optical properties of lens imaging. Then, full factorial experiments based on significant factors were performed to develop response surface models. The injection molding process window for optically optimized lenses was determined based on surface model and validation experiments. In 2020, Hanjui Chang et al. [10] proposed a method to optimize the warpage of parts by adding glass fiber into polymers, and analyzed the influence of glass fiber addition quantity and holding time on the forming warpage and deformation of structural parts. The results show that the warping deformation of parts can be reduced and the structural strength can be improved by adding glass fiber into the material; the more glass fiber is added, the better. In addition, the warping deformation and residual stress of injection products can be reduced by prolonging the holding time.

In addition, the artificial neural network can be used to optimize the process parameters of ICM technology according to the quality requirements of optical lenses (such as the precision of microstructure and geometric deviation), and the optimal process parameters can be obtained quickly. In 2019, Fei Guo et al. [11] developed an online decision system consisting of a new reinforcement learning framework and a self-predictive artificial neural network model for selecting appropriate process parameter settings to obtain optical lenses with high dimensional accuracy. In 2021, Mehdi et al. [12] used artificial neural networks and Taguchi technology to find an optimal set of process parameters. The analytic hierarchy process (AHP) was used to calculate the weight of each defect in the proposed thin-wall part. The results show that the error margin of the proposed optimization method is 1.5%, which is due to the uncontrollable influence over the injection molding process, and is acceptable. In 2020, M. S. Huang et al. [13] proposed a multi-layer perceptron (MLP) neural network model combined with quality indicators for the rapid and automatic prediction of finished product geometry. The quality indexes that are closely related to the quality of parts are extracted from the pressure curve, and then the MLP model is learned to predict the quality of products. The results show that the peak pressure index is highly correlated with the geometric width of the products. The first stage pressure holding index, pressure integral index, residual pressure drop index and peak pressure index with respect to geometric width were trained and tested accurately (over 92% accuracy), indicating that the method was effective.

In recent years, with the increasing requirements for precision control in production in the industrial field, and the promotion of industry 4.0, the application of sensing technologies in injection molding machines can enable users to more easily optimize the production process, ensure good production quality, and reduce the production cost and failure rate. Therefore, more and more sensors are being used to monitor and forecast the quality of injection molding products in injection molding machines. Gordon et al. [14] used multivariable sensors to model product quality for injection molding. The melt pressure and temperature are obtained by adding piezoelectric ceramic elements and an infrared thermoelectric reactor to the sensor, and the melt velocity is derived from the transient response of the melt temperature as the polymer melt flows through the sensor lens. The melt parameter data obtained from the sensor (cause) were combined with the quality indexes (results) of the finished part, such as thickness, width, length, weight and tensile strength; finally, a prediction model of the quality, size and structural characteristics of the part was obtained. M. S. Huang et al. [15] applied a sensor to the nozzle, flow and cavity to evaluate the quality of the melt pressure sensor; they performed Pearson correlation based on the three and the quality of the pressure signals of different positions and product weights were evaluated. The results showed the peak stress index and the quality of the products. Secondly, the energy and viscosity indexes were extracted from the pressure signals of the nozzle and runner, and the proposed monitoring system and quality indexes provide a convenient and effective means for monitoring the change in melt quality in the continuous injection molding process. In 2022, Hanjui Chang et al. [16] calculated the change in clamping force in the injection molding process by placing sensors on the large column of the injection molding machine, and carried out the real-time monitoring of product quality based on this. The reliability of the proposed method was evaluated for three different injection parameters (margin position, measurement endpoint and measurement time). In addition, the method was applied to the actual production of plastic bottle caps, and the weight of plastic bottle caps was taken as the evaluation index. The results show that the weight of plastic bottle caps can be controlled within the qualified range by using a continuous production mold. In other words, it is feasible to apply the change of clamping force to monitor the quality of injection molded products.

In addition, among the many process variables, cavity pressure is considered to be the “fingerprint” of injection molding, and it has a decisive effect on the final quality of the product. If it is possible to monitor the cavity pressure in real time, that is, by collecting, displaying and analyzing the cavity pressure during the molding process of plastic products, process optimization and quality inspection can be realized. M. S. Huang et al. obtained a pressure change curve form the sensor on the nozzle and the sensor in the cavity, and compared the injection pressure and injection speed in the filling stage with the V/P switching time, filling time, gate freezing time and pressure in the holding pressure stage. The parameters of pressure/time were optimized, and the detection and improvement of product quality were realized. M. S. Huang et al. found a strong correlation between the four properties of packing pressure, part weight, part thickness, and maximum cavity pressure. Additionally, part weight is highly dependent on packing pressure and is closely related to maximum cavity pressure. So, they used part weight as a quality criterion for quality control, and confirmed the feasibility of predicting part weight using the maximum cavity pressure value.

**Figure 1:**
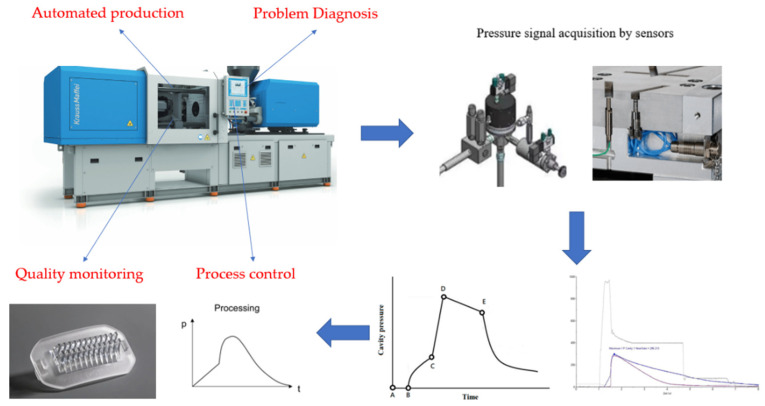
Application of pressure sensors in injection molding machines.

It can be seen that there is a close relationship between the cavity pressure and the final quality of the injection molded product. It can be judged whether the product is qualified by monitoring the characteristics of the cavity pressure curve of each production cycle. We no longer need to rely on statistical experimental methods, as we can use computer-aided simulations or operator experience to optimize and improve product quality. As shown in Figure 1, the scientific molding method is used to change the injection molding from “machine-centered” to “plastics perspective”, using various sensors to reveal the flow of molten resin in the machine and the mold to optimize the injection parameters, and then improve product quality for scientific validity.

Among the many process variables, cavity pressure is considered as the fingerprint of injection molding, which has a decisive effect on the final quality of the product. If the cavity pressure can be monitored in real time, that is, through the collection, display and analysis of the cavity pressure in the molding process of plastic products, it can be used for the realization of process optimization, quality testing and other functions. M. S. Huang et al. [17], through the placement of nozzle and cavity sensors, determined the pressure curve, the filling stage of the injection pressure and injection speed, V/P transformation time, sprue, the fill time, the freeze time, the pressure maintaining phase of the holding pressure/time parameter optimization, the realization of optimal product quality testing and the improvement of the quality of the products. M. S. Huang et al. [18] found a strong correlation between the four attributes of holding pressure, part weight, part thickness and maximum cavity pressure. In addition, part weight is largely dependent on the holding pressure and is closely related to the maximum cavity pressure. Therefore, they took part weight as the quality standard, and proved the feasibility of predicting part weight using the maximum cavity pressure value.

It can be seen that there is a close relationship between the cavity pressure and the final quality of the injection products, and the quality of the products can be judged by monitoring the characteristics of the cavity pressure curve in each production cycle. We no longer need to rely on statistical experimental methods, computer-aided simulations or operator experience to optimize and improve product quality. The method of scientific molding has enabled us to move, from “machine-centered” to “plastic perspective”, using a variety of sensors to reveal the flow of molten resin in the machine and mold to optimize the injection parameters, and then improve the quality of products scientifically and effectively.

Wavelet analysis has been proved to be a powerful method in the signal processing of fault diagnosis. Analyses include data compression and representation, as well as signal denoising. Discrete wavelet transform (DWT) has the advantage of time-frequency representation shown by Fourier transform, which can be used for the pattern recognition and feature extraction of fault diagnosis in industrial process. T. Mohanraj et al. [19] obtained vibration signals through sensors, used wavelet transform extraction to extract Holder index as features, and used various machine learning algorithms (such as SVM, DT, KNN, KB and MLP) to predict cutter state, while also monitoring milling cutter side wear. The analysis results showed that, DT and SVM have the highest accuracy of 100% and 99.86%, respectively. Sukhjeet Singh et al. [20] introduced a comprehensive method for the fault detection and classification of rolling bearings in rotating machinery. To compare the feasibility of the proposed method, they compared two kinds of amplification, one is the combination of permutation entropy (PE) and flexible analytic wavelet transform (FAWT), and the other is the integration of binary discrete wavelet transform (DWT) and PE. The above two methods are used to decompose the signals of healthy and faulty bearing systems under different working conditions. The PE values of the decomposed signals are calculated and then input into the support vector machine (SVM) classifier as feature vectors to classify different types and sizes of fault. The classification results of the two methods have been compared. The results show that FAWT integrated PE is more effective and robust than DWT integrated PE in bearing fault detection and classification.

In 2022, Hanjui Chang et al. [21] used the multi-objective optimization method to optimize the injection molding defects of automobile pedals. Aiming at the factors influencing the optimization process (mold filling time, mold filling pressure, melt temperature, cooling time, injection time, etc.), the warping deformation was analyzed by flow simulation, and the warping parameters and cycle time were assessed by setting different cooling distribution, pressure and temperature schemes. The multi-objective optimization design is mainly used to describe the relationship between cycle time and warpage, and the Pareto boundary of cycle time and warpage is used to identify the deviation function and radial basis function network. In 2022, Hanjui Chang [16] et al. proposed a real-time product quality monitoring system based on the change of clamping force during injection molding. The change of mold locking force can reflect the change of molding pressure in the injection process and further map the change of injection parameters. Therefore, in the research on three different injection parameters (position allowance, measuring end point and measuring time), to assess the reliability of the proposed method, the results show that by changing the clamping force index and the corresponding injection parameters’ calibration model integration, we can mold over a continuous production time and control the weight of the plastic bottle cap in the qualified range of products.

Generally speaking, the influencing factors between different units encountered in the experiment cannot be directly compared; for example, apples and bananas, although they are also fruits, cannot be compared between different varieties. Therefore, we have introduced a method of identification and evaluation to solve this problem. Through the simple method of signal-to-noise ratio, the influencing factors of different units, such as temperature, pressure, and length, are transformed into identifiable factors for comparison. A large number of studies show that the identifiable evaluation method is very effective and feasible for injection molding production.

Although wavelet transform is widely used in pattern recognition and fault detection, it is seldom used in injection molding machines. For the signal obtained from the sensor, wavelet analysis can be used for noise reduction and feature extraction, which means wavelet analysis can be connected with the injection molding machine through the bridge of sensing technology. Wavelet analysis can be used to realize signal noise reduction and feature extraction, but it cannot compare different factors. Although it can identify and compare factors between different units, it lacks means for signal analysis and feature extraction. Therefore, if wavelet analysis and other recognizable methods can be combined, the problem of cavity pressure analysis can be solved, and this can provide numerical analysis results for technicians, which will be very beneficial to the injection molding production process.

**Figure 2:**
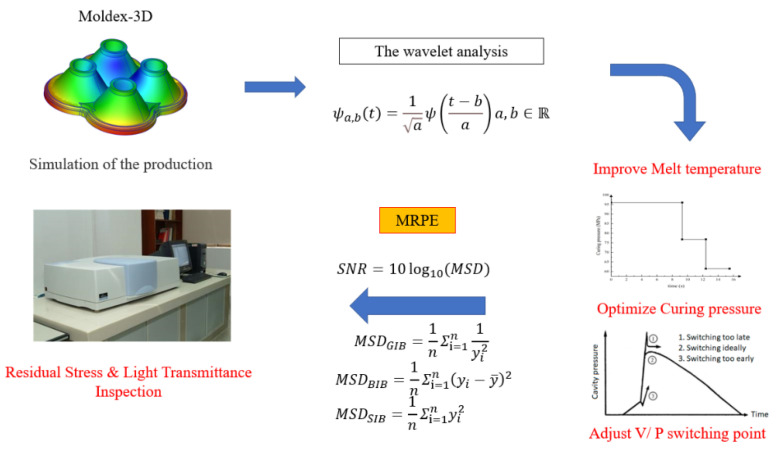
Concept for optical components experimental.

As shown in Figure 2, the purpose of this study is to propose a method for monitoring the quality of injection molded parts. Taking the injection process of an optical component as an example, a sensor and data acquisition system were used to select and obtain the process variables most closely related to product quality from the process, and wavelet transform was used to reduce noise and extract features of the signal. In addition, the effects of V/P switching point and melt temperature on the transmittance and volume shrinkage of the lens are also investigated.

## 3. Materials and Methods

### 3.1. LSR Materials

**Figure 3:**
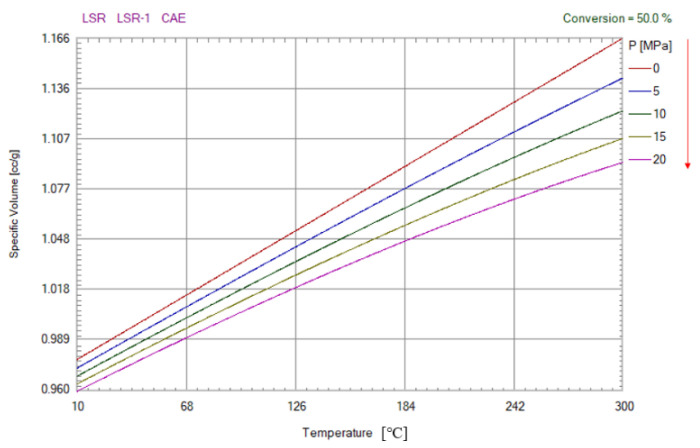
PVT curve of model LSR-1 (LSR material is manufactured by CAE).

The material used in this study is LSR, with model LSR-1 produced by the CAE manufacturer. The PVT curve of this material is shown in Figure 3. Compared with PMMA and PC, LSR has many advantages such as heat and weather resistance, non-toxicity, electrical insulation, biocompatibility, colorlessness and transparency, tear resistance and so on. It is widely used in medical, electronic and electrical, food and maternal and infant product industries. In the field of electronic packaging, liquid silica gel is mainly used for the potting of electronic products. Electronic packaging liquid silica gel can play a role in sealing, waterproofing, dustproofing, thermal conductivity, shockproof and the insulation of electronic products. In addition, LSR material is also used as the encapsulation glue of LEDs, and its products have the characteristics of high transparency and high refractive index. Table 1 shows the comparison of optical properties of three commonly used optical rubbers, PC, PMMA and LSR.

### 3.2. Wavelet Transform

The traditional signal theory is based on Fourier analysis, but Fourier transform, as a globally used approach, has some limitations, such as not having the ability of local analysis, or to analyze non-stationary signals. Researchers resolve this limitation by making various modifications to the Fourier transform, such as STFT (Short Time Fourier Transform). However, as the sliding window function adopted by STFT is fixed once it is selected, it is not accurate in the time domain of high frequency signals and low frequency signals, and does not have an adaptive capacity. Therefore, we introduce the concept of wavelet transform to solve this problem.

Compared with Fourier transform, wavelet transform is a time and frequency domain local transform that can effectively extract information from the signal. Wavelet transform can use scale parameters and translation parameters to change the shape of the window size, so that the signal can derive an accurate time resolution with high frequency and accurate spatial resolution with low frequency in multiscale analysis. This helps solve many difficult problems that cannot be solved by Fourier transform. The wavelet function is shown in Equation (1):

(1) $$\psi_{a,b}\left(t\right)=\frac{1}{\sqrt{a}}\psi \left(\frac{t-b}{a}\right)a,b\in \mathbb{R}$$

$\psi_{a,b}\left(t\right)$ is the wavelet function, *a* is the scale parameter, *b* is the translation parameter. When *a* > 1, the modified basis function is stretched on the basis of the original wavelet, thus representing the low frequency characteristics. When *a* < 1, the changed basis function shrinks somewhat on the basis of the original wavelet, which is expressed as the high frequency characteristic.

### 3.3. Multiple Recognizable Performance Evaluations

Multiple Recognizable Performance Evaluation is based on the Taguchi method of Dr. Genichi Taguchi. The Taguchi method aims to use the function relations of quality, cost and benefit, and experimental design in statistics in the process of product design, so as to reduce the number of tests, save the manpower and material resources required, develop high-quality products, and achieve the maximum benefits. However, the same physical experiments are not available in the identification of defect factors (such as no unit defect factor, different units of defect factor and interaction of defective factor), because the corresponding data are based on different physical properties or units, so they cannot simply be carried out via the traditional physical experiments, comparison and contrasts. Take entropy, temperature and length, for example. They employ different units, so we cannot compare them directly. Therefore, we propose a multiple identifiable performance evaluation method for the iterative calculation of the post-process to determine the common operability of different physical properties and to propose the basis for optimization. In addition, if there are multiple interactive independent variables in the experimental study that is, when one variable changes, other independent variables also change the multiple identifiable performance evaluation method and variable separable model can be combined to realize the identifiable evaluation of different defect factors. The identifiable performance evaluation method and variable other models can be combined to realize the identifiable evaluation of different defect factors.

The influence of process parameters on the quality of optical liquid silicone rubber lenses was analyzed and compared. In the optimal parameter analysis, the experimental results were transformed for the evaluation of the characteristic values using the signal-to-noise ratio, rather than the average value. The formula for calculating the signal-to-noise ratio is shown in Equation (2):

(2) $$\frac{S}{N}=-10\log\left(MSD\right)$$

where *MSD* is the mean square deviation of the output characteristics. When the quality characteristics are continuously subjected to engineering analyses, the signal-to-noise ratio characteristics can be classified into three types: the smaller the better, the more standard the better, and the larger the better. The research goal is to determine the best design parameters for high-quality optical liquid silicone rubber lenses. In order to maximize the light transmission, the larger the signal-to-noise ratio the better; in order to standardize the specification of injection metrology, that is, to control the final dwell position of the injection screw, the smaller the signal-to-noise ratio the better; in order to minimize warpage and residual stress, the smaller the signal-to-noise ratio the better. As shown above, the quality characteristics of “the larger the better”, “the more standard the better” and “the smaller the better” were used in this study. These three quality characteristics can be expressed in *MSD* as Equations (3)–(5):

(3) $$MSD_{GIB}=\frac{1}{n}\Sigma_{i=1}^{n}\frac{1}{y_{i}^{2}}$$

(4) $$MSD_{BIB}=\frac{1}{n}\Sigma_{i=1}^{n}{\left(y_{i}-\bar{y}\right)}^{2}$$

(5) $$MSD_{SIB}=\frac{1}{n}\Sigma_{i=1}^{n}y_{i}^{2}$$

where $y_{i}$ is the measured value for the i sample and n represents the total number of samples. Because -log is a monotonic decreasing function, the S/N value should be maximized. Thus, the S/N values are calculated using Equations (3)–(5).

**Figure 4:**
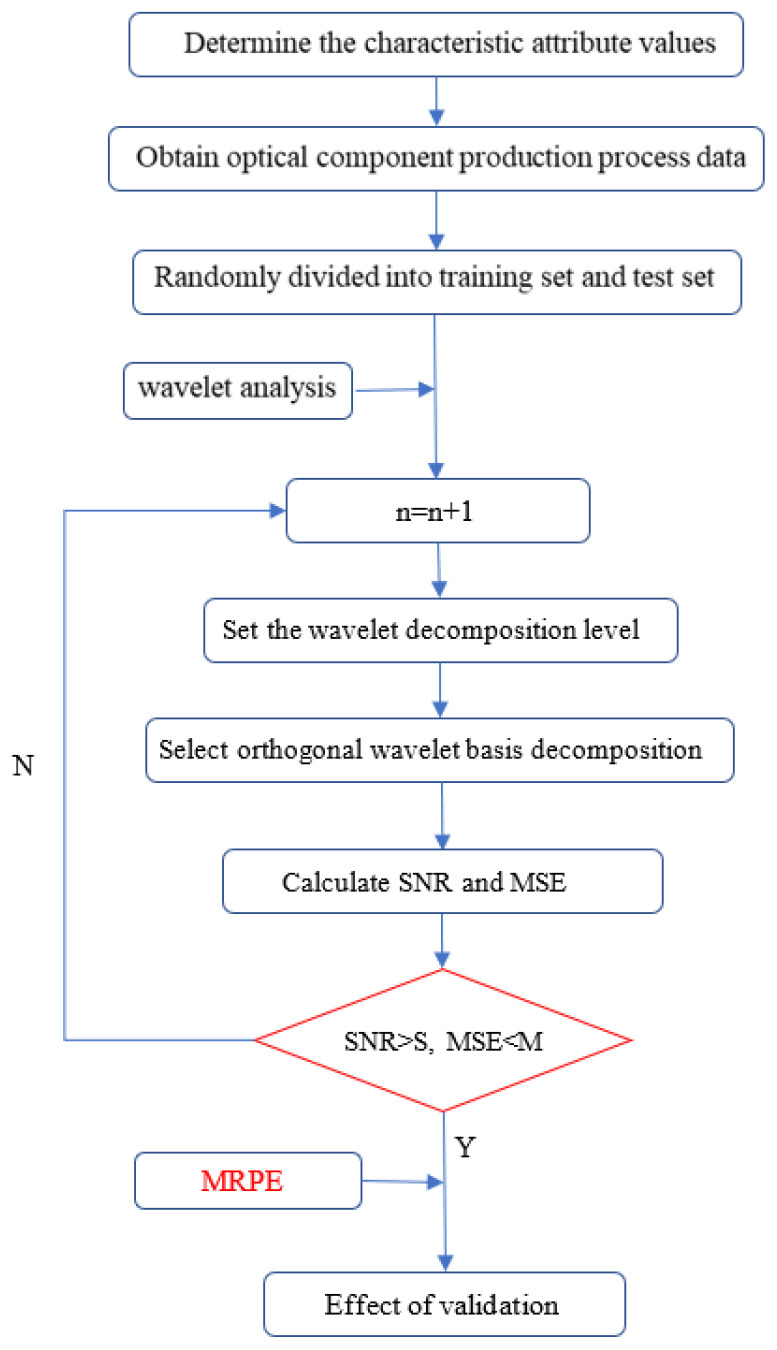
Method flow chart.

Figure 4 shows the analysis process for the quality optimization of optical components. In this paper, the entire analysis process starts with the confirmation of the values of the characteristic attributes, followed by the sampling of the optical lens production process data. First, the data results are analyzed in groups, which facilitates subsequent comparative experiments. Next, parameters such as orthogonal wavelet bases, wavelet decomposition levels, threshold selection rules, and threshold quantization functions are determined, followed by the calculation of two evaluation criteria, signal-to-noise ratio (SNR) and mean square error (MSE). Finally, the effect is verified by applying identifiable methods. The goal of this stage is to find the best process parameters with maximum probability.

## 4. Case Study

**Figure 5:**
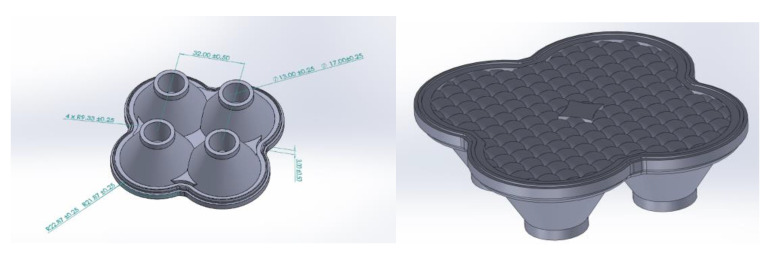
Auto LED lens sample diagram by SolidWorks.

**Figure 6:**
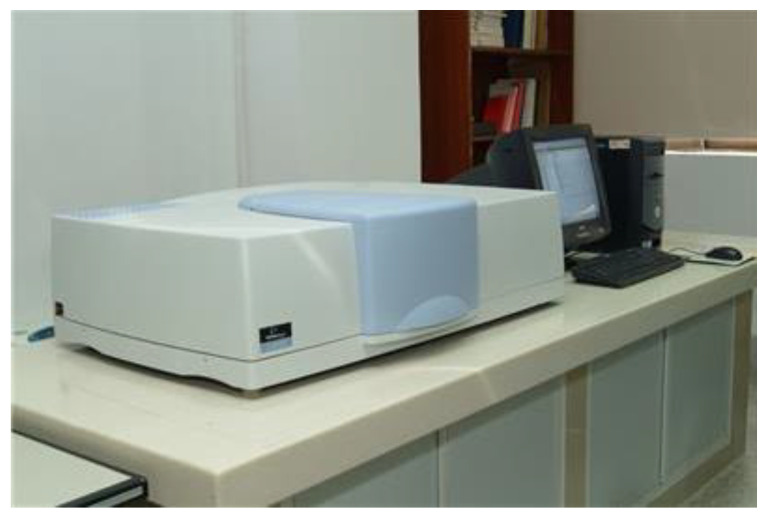
Lambda 950 UV/VIS Spectrometer.

In this study, we used a liquid optical silicone component for automotive applications, which is 77.74 mm × 77.74 mm × 23 mm in size, 22.483 mm in maximum flesh thickness, 0.101 mm in minimum flesh thickness, and 53972.35 ${\mathrm{mm}}^{3}$ in volume. In this case, study, SolidWorks software was first used for modeling. In the SolidWorks software, the model is built by stretching, rotating and arraying functions, so that it can fully express the structural characteristics of the component, and finally the liquid optical silicone component model for vehicles was established, as shown in Figure 5. The Moldex3D software was used to perform the computer simulation of silicone lens molding. In addition, the experiments were conducted on an all-electric injection molding machine (KM50-250 PX, Krauss Maffei, Shanghai, China). For the actual output, the transmittance tests were performed using a Lambda 950 UV/VIS Spectrometer, with the model number shown in Figure 6. The PerkinElmer lambda 950 spectrophotometer is characterized by high sensitivity, high stability, and easy operation; it has a spectral range 190–3000 nm, double beam, and Double monochromator. Its bandwidth is either 0.5, 1, 2 or 4 nm, the stray light is <0.01T, the wavelength accuracy is +0.1nm, the wavelength repeatability is <0.05 nm, photometric accuracy +0.003A, photometric repeatability <0.001 A, baseline drift <0.0001 A/h, limit flat +0.001A, and noise <0.00008 A. The lambda950 Diffuse reflection/transmittance system equipped with a 150mm integrating sphere: can be used for the testing of liquid and solid powders; with transmission and reflection measurement modes. It has variable temperature test functions in the region of 100 °C.

The optical lens has high quality requirements. The difficulty lies in precisely controlling the geometric accuracy, optical performance and the forming accuracy of the optical surface microstructure, reducing the processing cost and improving the production efficiency. Due to the inherent characteristics of polymer materials, such as large thermal expansion and contraction effect, molecular orientation and birefringence, it is difficult to meet the high-quality requirements of optical lenses via the ordinary injection molding process. In addition, on the surface of the polymer optical lens, the size of such microstructure is in the micrometer or even nanometer range. In addition, due to the uneven wall thickness of the optical plastic lens, the density and refractive index distribution of the plastic lens molded by the ordinary injection method are uneven, and the residual internal stress and birefringence phenomenon are generated in the lens. The surface accuracy cannot meet the use requirements. If mold designers and technologists can determine in advance the shear rate, temperature, pressure and time of molten material flowing through the pouring system and mold cavity, they can reasonably determine the mold structure and injection process, and improve the primary success rate of the product. It is also the ultimate purpose of this study to adjust the process parameters and improve the quality and yield of the lenses.

Optimize the V/P switching point: The correct setting of the switching point is a very important process in injection molding production. If the pressure is too low, too much melt will be pushed into the cavity by the holding pressure, which will give rise to a lack of material. If the holding pressure is too high, the product will be compressed too much, forming parts with flying edges and high internal stress, and the mold release will not be stable enough, increasing the weight of the product.

Optimize the melt temperature: The melt temperature has a great influence on the molding quality and molding efficiency of the rubber parts. If the melt temperature is high, the fluidity of molten rubber is better, which enables the rubber to stick to the cavity and develop a high-quality surface, but it will make the rubber curing time longer and make the mateieral more easy to deform when ejecting, which is better for the crystallization process of crystalline rubber and prevents the size change of rubber parts in storage and use; if the melt temperature is low, it is difficult for the molten rubber to stick to the cavity, which will lead to an increase in internal stress, surface lusterlessness, silver lines and fusion marks. If the melt temperature is low, the melt will struggle to adhere to the cavity, resulting in increased internal stress and a non-glossy surface.

We conducted a two-factor full analysis of the experiment. Table 2 shows the process parameters for optical lens injection molding with the V/P switching point position ranging from 94 to 98 and a melt temperature from 20 to 30 °C. This study explores the correlations and outliers between quality indicators and quality (residual stress and optical transmittance) for a wide range of process parameter adjustments.

## 5. Discussion

A process parameter optimization method based on light transmittance and part weight deviation is proposed in this paper. Because the operation of measuring the weight and light transmittance of each part after it is ejected from the cavity is very time consuming, this study proposes an alternative method to estimate the mass of the part. As described in the literature review section, the cavity pressure curve provides a reliable basis for product quality stability, which can provide a convenient and reliable method for ensuring part quality under various processing conditions. Therefore, we investigated the possibility of using the cavity pressure distribution to predict the mass of each lens ejected from the mold.

Most of the injection molding process is determined by position pressure, but sometimes the precise position pressure is difficult to control. If the pressure transfer point is set too low, the cavity pressure will spike, because the cavity pressure will not have reached the value of pressure transfer during the injection, and it then enters the pressure preservation stage, resulting in the mutation of cavity pressure to form a spike. If the position of the pressure transfer point is set too late, the cavity will be almost filled during the injection process, and the pressure transfer point will mainifest a concave peak, that is to say, the pressure curve will show a double peak phenomenon when the pressure transfer point is set too late. As shown, the cavity pressure signal mutates at the V/P transition point, which is a singular point with discontinuous derivatives. Therefore, judging whether the V/P transition point is reached can be altered into detecting whether there are singular points in the pressure measurement data. There are many methods of singularity detection, among which wavelet transform uses the time window and frequency window, and has good local characteristics in the time and frequency domains as well as an ability to adap to the signal, so it is an effective method for analyzing the singularity of the non-stationary signal. Based on the singular value detection principle of wavelet transform and the actual characteristics of injection molding production, a real-time detection method of the V/P switching point based on wavelet transform is presented. Firstly, the wavelet analysis is carried out on the pressure data of the first batch of mold cavity, and the information of wavelet decomposition depth and transformation domain is obtained. Then, wavelet analysis and domain value discrimination are applied to the data in the sliding window to realize the online detection of the V/P switching points.

To realize the wavelet noise reduction processing of cavity pressure, the wavelet toolbox or wden function in Matlab software is used for noise reduction processing. According to the three steps of wavelet, processing parameters such as orthogonal wavelet basis, wavelet decomposition level, threshold selection rule and threshold quantization function need to be determined. Only the effect of the type of orthogonal wavelet basis on noise reduction is studied here. The wavelet decomposition level can be set to 3, because the presence of too many layers will reduce the similarity with the original signal, and if the number of layers is too small, the noise reduction effect of the signal will not be obvious. For threshold selection, heuristic thresholding can be used; because extreme threshold estimation and unbiased likelihood estimation tend to be conservative, resulting in incomplete denoising. For the threshold quantization function, soft thresholding is used; this is because soft thresholding makes the denoised curve smoother. The choice of orthogonal wavelet bases needs to be determined by the comparison of the noise reduction effects. Here, we compare and select several common wavelet bases, such as haar, db4, db8, sym4 and sym8.

**Figure 7:**
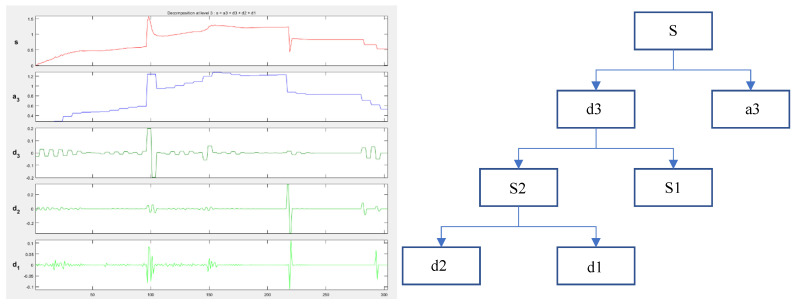
One-dimensional wavelet decomposition.

The feature extraction of cavity pressure aims to provide a greater basis for data analysis and quality monitoring of the system, and wavelet packet decomposition provides a method for extracting features in the time-frequency domain. After the signal is decomposed by N layers of the wavelet packets, 2^N^ orthogonal frequency bands can be generated, and the sum of energy in different bands is equal to that of the original signal. The decomposition of the wavelet packet at three scales is shown in Figure 7, where the original signal s is decomposed into four signals a3, S1, d1 and d2. In addition, this study evaluates the noise reduction performance via two evaluation criteria: signal-to-noise ratio (SNR) and mean square error (MSE).

In the processing of the cavity pressure curve, the cavity pressure data are denoised based on wavelet change and cavity pressure feature extraction is performed based on wavelet packet decomposition. The wavelet denoising process can be divided into three steps: wavelet decomposition, threshold processing and wavelet reconstruction. In addition, SNR and MSE are used to evaluate noise reduction performance.

SNR is calculated as shown in Equation (6):

(6) $$SNR=10\log_{10}\frac{P_{s}}{P_{n}}$$

MSE is calculated as shown in Equation (7):

(7) $$MSE=\frac{1}{N}\overset{N}{\underset{n=1}{\sum }}{\left[F\left(n\right)-f\left(n\right)\right]}^{2}$$

$P_{s}=\frac{1}{N}\overset{N}{\underset{n=1}{\sum }}f^{2}\left(n\right)$ expresses the original signal power, $P_{n}=\frac{1}{N}\overset{N}{\underset{n=1}{\sum }}{\left[F\left(n\right)-f\left(n\right)\right]}^{2}$ expresses the noise power of the inclusion in the original signal, F(n) expresses the noise signal, and f(n) is expresses the original signal.

The larger the SNR is, the more obvious the denoising effect is, and the curve after denoising will be smooth. The mean square error represents the difference of in the signal before and after denoising. The smaller the difference is, the more similar the signal curve after denoising will be to the original signal curve.

**Figure 8:**
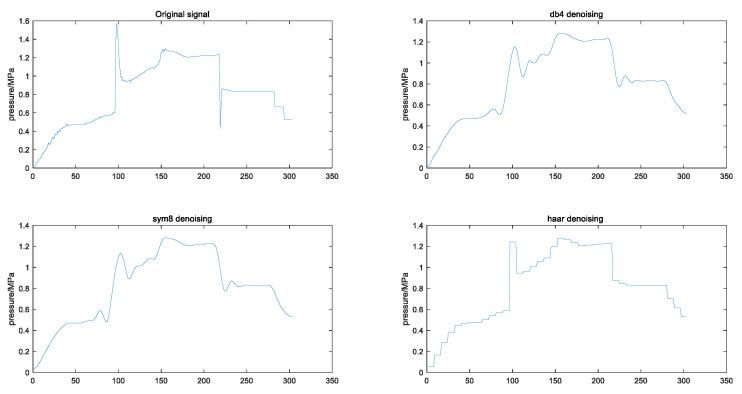
Comparison of the denoising effect of several commonly used wavelet bases.

Figure 8 shows the curves of the original signal after noise reduction with three different wavelet bases, db4, sym8 and haar, and it can be visualized that the curves are smoother after processing with db4 and sym8 wavelet bases. In addition, we calculated the signal-to-noise ratio and mean square error values after processing with different wavelet bases, as shown in Table 3, which shows the signal-to-noise ratio and mean square error in the case of noise reduction with the commonly used wavelet bases haar, db4, db8, sym4, and sym8. The principle to be emphasized is that the larger the signal-to-noise ratio, the smoother the noise reduction, the smaller the mean square error, the more similar to the original curve, and the more information retained. Haar is not smooth enough, sym4 has the best effect, db4 and sym8 have the second-best effect. Sym4 has the best effect on the pressure signal-to-noise ratio index, and the other indexes have the second-best effect. Sym4 has a better noise reduction effect than other wavelet bases.

**Figure 9:**
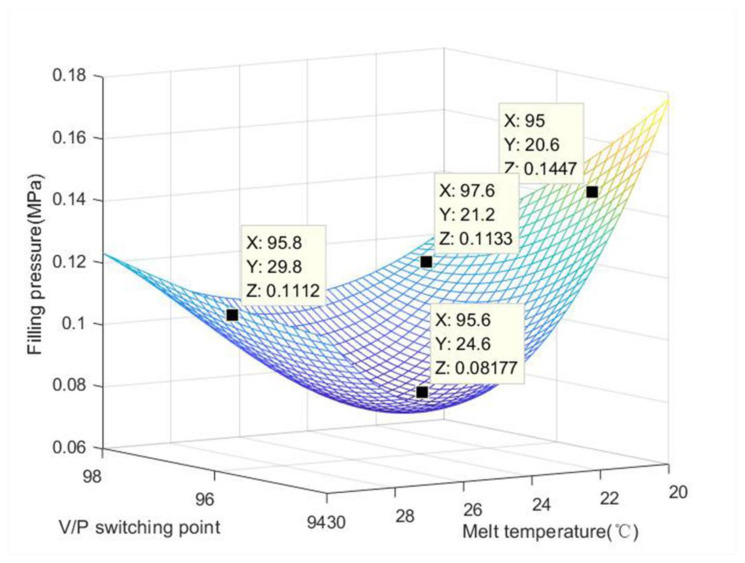
Effect of melt temperature and V/P switching point on the filling pressure.

Melt temperature has a great influence on the molding process, molding properties, molding conditions and physical and mechanical properties of plastics. Generally, with the increase in temperature, the melt viscosity decreases, the pressure in the barrel, nozzle and mold pouring system decreases, and the melt flow length in the mold increases, thus improving the forming energy. The injection rate increases, the melt time and mold filling time decrease, the injection cycle is shortened, and the surface finish of the product is improved. However, when the temperature is too high, plastic easily undergoes thermal degradation, and the performance deteriorates. It can be seen from Table 4 that a too large V/P switching point is unnecessary. Our optimization study has shown that it only needs to be in 96–98. The main factor that affects the change in gate pressure is melt temperature. When the melt temperature increases, the gate pressure decreases. When the melt temperature is 30 °C, the pressure value is 1.261 MPa, and when the melt temperature is 20 °C, the pressure value is 1.572 MPa. In addition, it can be seen from Figure 9 that the three-dimensional plane has a peak-valley shape, which also means that the filling pressure at the lowest point is the smallest, which can provide a basis for the engineer to set the filling pressure.

**Figure 10:**
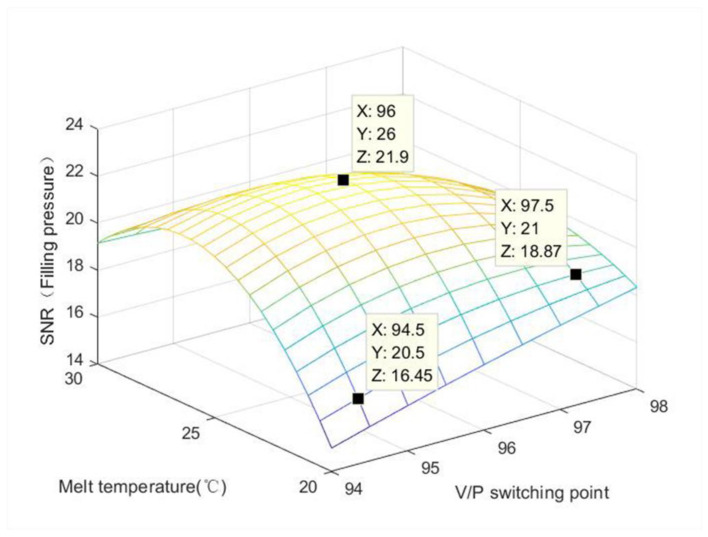
Effect of melt temperature and V/P switching point on the filling pressure signal.

The filling pressure is a very important indicator in the injection molding process. The pressure determines the size of the injection molding process window, and the distribution and transfer of pressure in the mold cavity affects the appearance and dimensions of the product. Table 5 shows that when the V/P switching point is 96, the filling pressure signal is larger, and when the V/P switching point is 94, the filling pressure signal is smaller. From Figure 10, it can be seen that when the melt temperature is 26 °C and the V/P switching point is 96, the signal value of filling pressure is the largest.

**Figure 11:**
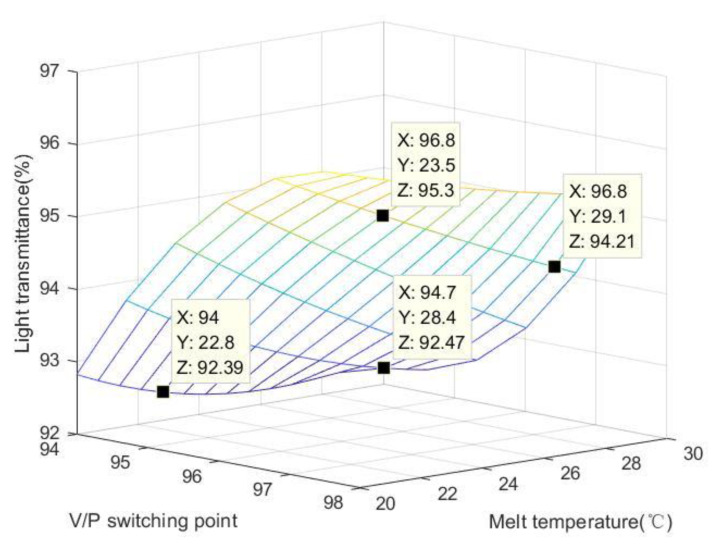
The relationship between V/P switching point, light transmittance and melt temperature.

Table 6 shows the average residual stress value of each component under the influence of V/P switching point and melt temperature change. After visualization of the results, it can be seen from Figure 11 that with the increase in melt temperature, the overall residual stress of the workpiece decreases, especially in the central region. When the melting temperature is 20 °C, the residual stress is larger. When the melt temperature is between 20 °C and 30 °C, the residual stress distribution mainly concentrates near the gate edge and decreases along the melt flow direction. When the melt temperature is 20 °C, the low melt temperature leads to the insufficient Brownian motion of molecular chain segments after melt filling, and the molecular chain segments freeze in the reverse direction, resulting in large residual flow stress. The lower temperature of the mold intensifies the cooling of the parts, and the shrinkage inconsistency produces large thermal residual stress. The combination of the two results in high residual stress throughout the workpiece. With the increase in melt temperature, the degree of direction of molecular chain segments increases and the flow residual stress decreases during the cooling phase of the melting. The increase in melt temperature slows the cooling rate of the parts and reduces the residual thermal stress. Therefore, the residual stress decreases with the increase in melt temperature.

**Figure 12:**
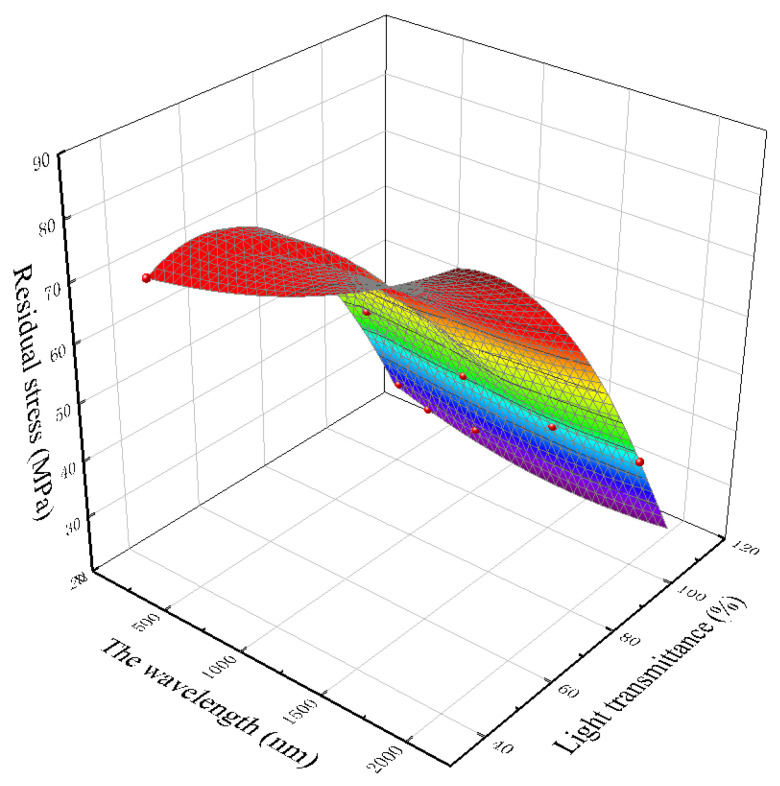
The relationship between residual stress and the transmittance of light at various wavelengths.

Residual stress is the phenomenon of the surface warping and deformation of injection molded parts due to the stress remaining inside after demolding during polymer processing, and it especially occurs during injection molding. The residual stress of injection molded products usually leads to warpage and deformation, resulting in shape and size errors. At the same time, crazing and other defects caused by residual stress will cause the premature failure of components during use, affecting their serviceability. There are two sources of residual stress in injection molded products: orientation residual stress and shrinkage residual stress. For the injection molding process, the melt injection temperature, mold wall temperature, melt filling time and filling speed, pressure holding pressure and length of the runner will affect the flow stress. Residual stress is an important index that affects the transmittance of the polymer lens. From Figure 12, we can see that the residual stress is negatively correlated with the light transmittance. The larger the residual stress is, the smaller the light transmittance is. For precision optical lenses, the greater the residual stress is, the smaller the transmittance of light in different bands is, and the overall performance will be limited.

**Figure 13:**
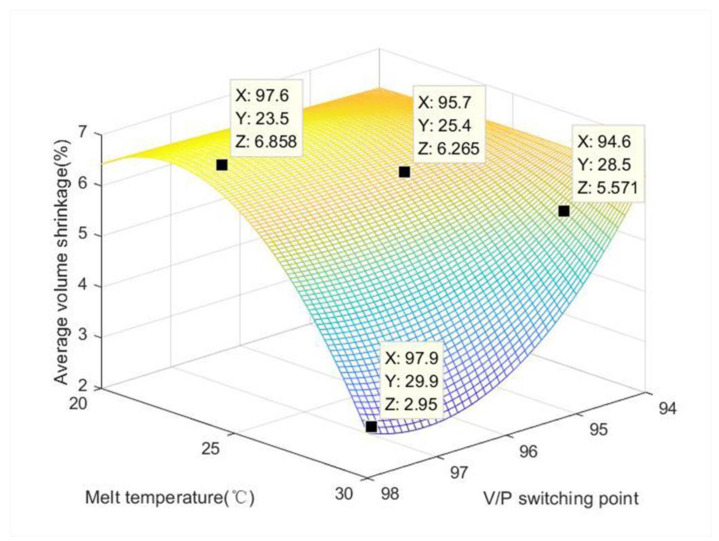
The average shrinkage of components as a function of V/P switching point and melt temperature.

Usually, the volume shrinkage is expressed as the percentage change in volume when the high temperature and pressure states are reduced to normal temperature and pressure. Positive values represent volume shrinkage and negative values represent volume expansion, which may be due to over-pressurization. The difference in shrinkage throughout the plastic part and profile causes internal residual stresses, the effect of which is exactly the same as that of external forces, while the results show that the distribution of residual stresses in the same product is closely related to the optical properties, which are themselves greatly influenced by residual stresses. The locations of the residual stress can give rise to poor optical performance, and vice versa. In other words, the uneven volume shrinkage will not only lower the dimensional accuracy of the product, but will also indirectly affect the optical properties of the product. In addition, Table 7 shows the volume shrinkage of the product with different parameter gradients. Due to the characteristics of LSR, the warpage and volume shrinkage are very small. Therefore, the change in the volume of the product before V/P switching and after V/P switching represents the amount of carbon emission, which is equivalent to the injection molding fingerprint. If the change parameters of the process are not adjusted properly, then and more volume shrinkage will occur, which will cause an increase in carbon emissions. As shown in Figure 13, when the V/P switching point is 98 and the melt temperature is 30 °C, the product displays the best quality and the smallest volume shrinkage with a maintenance of size of 2.895%, which also leads to the lowest carbon emission.

According to the above results, wavelet transform can be used for real-time detection of V/P switching points. Through the wavelet analysis of the pressure data of the first batch of the mold cavity, the depth of wavelet decomposition and the value of the transform domain are obtained. Then, wavelet analysis and domain value discrimination are applied to the data in the sliding window to realize the on-line detection of the V/P switching point. In addition, wavelet analysis theory mainly focuses on wavelet transform and wavelet packet decomposition. As the first step of pressure data processing, the pressure data are denoised, and the wavelet basis is used for wavelet denoising. As the second step of pressure data processing, the energy characteristics of the wavelet packet nodes and some time-domain features are extracted, and the time-domain features include statistical features and empirical features. In addition, the melt temperature has a great influence on the residual stress of the product, and the residual stress is negatively correlated with the transmittance. The higher the residual stress, the smaller the transmittance. This result is consistent with the experimental results of Lin, Chao-Ming et al. and Tsai, K. M. The results of Lin, Chao-Ming et al. show that the filling pressure dominates the warpage effect of the lens and the melt temperature dominates its residual stress, while the results of Tsai, K. M. et al. show that the main factors affecting the optical properties of the lens are mold temperature, melt temperature, and cooling time. For precision optical lenses, the greater the residual stress, the smaller the transmittance of light of different wavelength bands, and the darker overall outcome will be. This is because the shear stress on these parts of the element is high during the flow process, so the molecules here are more oriented in the flow direction, and the degree of crystallization is increased. The transmittance decreases due to the high crystallinity. However, if the melt temperature is properly increased, the degree of molecular chain segment de-orientation will be increased and the flow residual stress will be reduced in the melt cooling stage, which can effectively prolong the formation time of the condensation layer of the workpiece, release a fuller flow residual stress on the surface of the workpiece, and thus reduce the average residual stress on the surface of the microlens array. Therefore, the residual stress of the workpiece as a whole shows a downward trend with the increase in melt temperature. It is worth noting that the increase in melt temperature is often accompanied by longer cooling time and more carbon emissions.

## 6. Conclusions

The different degree of residual stress in polymer lenses are caused by the improper design of the product or mold, the improper control of forming parameters, and other reasons. The residual stresses in polymer lenses can either be present at higher or lower levels, which can also adversely affect the appearance, mechanical properties, and optical properties of the products. Therefore, residual stress has always been the focus of scholars and industry, and it has important economic benefits that will improve the internal quality and external performance of transparent injection parts. This study aims to apply wavelet analysis techniques and multiple identifiable performance evaluation methods in the quality inspection of the injection molding machine. The results are as follows:

(1) In the lens injection molding experiment, the three key features of filling point, maximum pressure, and inflection point pressure are extracted from the cavity pressure signal in the monitoring process through the wavelet analysis toolbox of MATLAB. Combined with the singularity detection principle of the wavelet transform, the online detection of the injection pressure that can maintain the switching point is realized, which aids the injection production process in which the pressure increment of the mold cavity undergoes obvious changes at the injection pressure maintaining the switching point. Then, a diagnosis model of lens injection molding based on RSM can be established using these characteristics. In addition, the response surface function of lens transmittance is established using the extracted features. This method has the characteristics of the minor influence of the process parameters, a low signal-to-noise ratio, and a high degree of automation.

(2) When the melt temperature is 20 °C, the overall residual stress is larger. When the melt temperature is between 20 °C and 30 °C, the residual stress distribution of the workpiece is mostly concentrated in the edge area near the gate and shows a downward trend along the melt flow direction. In addition, the residual stress is negatively correlated with the transmittance. The greater the residual stress, the smaller the transmission. For precision optical lenses, the greater the residual stress, the smaller the light transmittance of different wavelength bands, and the better the overall performance. This is because, during the flow process, these parts of the element are subjected to a large shear stress, which makes the molecules here more aligned along the flow direction, so the degree of crystallization increases. Because of the high crystallinity, the light transmittance is reduced, so the final outcome appears dark.

(3) In this paper, when the V/P switching point was 98 and the melt temperature was 30 °C, the volume shrinkage was the smallest, and the size was 2.895%, which also means that the carbon emission would be the smallest. We prove the feasibility of developing fingerprints from the cavity pressure signal of injection molding system. The fingerprint is effectively developed by using wavelet analysis and multiple recognizable methods. This method is effective for the monitoring, diagnosis, and control of the injection molding process. The difference between the volume of the original hot melt adhesive entering the mold cavity and the volume of the component after molding is the volume change of the fingerprint. Usually, the volume change of this fingerprint is regarded as the source of carbon emission. As long as this change of volume is reduced, the carbon emissions resulting from the injection molding process will be lower. In general, raising the temperature is conducive to injection molding production. However, after the molding is completed, it must be cooled by the application of water, which causes carbon pollution. These fingerprints can be used to monitor and control the degree of carbon emissions resulting from the process, and researchers can effectively reduce carbon emissions by employing appropriate compensation or corrective measures.

Identifiable analysis and wavelet analysis offer an innovative method to optimize the distribution of identifiable parameters. In the past, evaluation relied on mold flow simulation analysis software and factory engineers blindly adjusting machines with limited effects, but both identifiable analysis and wavelet analysis methods can effectively the quantify configuration parameters used in injection molding to improve the stability, and thus improve the reliability and quality output, of injection-molded products.

## Figures and Tables

**Table 1:** Comparison of three optical polymer materials.

Performance	Unit	LSR	PC	PMMA
Birefringence	-	1.384	1.586	1.491
Density	g/cc	1.12	1.2	1.19
Saturated water absorption	%	0.1	0.2	0.3
Linear expansion coefficient	K-1	6 × 10^−5^	7 × 10^−5^	8 × 10^−5^
Transmittance	%	95	88–90	92
MFI	g/10min	10	7–25	0.5–16

**Table 2:** Material and Processing Parameters (LSR).

Material		Specification	Manufacturer
liquid silicone rubber		LSR-1	CAE
Temperature parameters		Unit	
Melt temperature		°C	20, 25, 30
Mold temperature		°C	160
Room temperature		°C	25
Filling parameters		Unit	
V/P switching point		-	94, 96, 98
Injection pressure		MPa	250
Holding parameters		Unit	
Holding pressure	1st stage	MPa	90
	2nd stage	MPa	70
	3rd stage	MPa	60
Holding time	1st stage	s	9
	2nd stage	s	3
	3rd stage	s	3
Cooling parameters		Unit	
Cooling time		s	30

**Table 3:** Comparison of the denoising effect of several commonly used wavelet bases.

	Db4	Sym4	Haar	Sym8
SNR	21.2116	23.2306	23.6309	21.4655
MSE	0.076734	0.060819	0.05808	0.074524

**Table 4:** Influence of pressure change at the gate.

V/P Switching Point	Melt Temperature/°C	Filling Pressure/MPa	Gate Pressure/MPa
94	20	0.179	1.572
94	25	0.145	1.480
94	30	0.121	1.261
96	20	0.090	1.572
96	25	0.081	1.480
96	30	0.104	1.261
98	20	0.110	1.572
98	25	0.115	1.480
98	30	0.123	1.261

**Table 5:** Control factor and signal-to-noise ratio.

Control Factor		Signal Noise Ratio	
V/P Switching Point	Melt Temperature/°C	Filling Pressure	Center Temperature
94	20	15.00567	−28.2296
94	25	16.80132	−27.9050
94	30	18.32873	−27.5676
96	20	20.87042	−28.2371
96	25	21.86981	−27.9116
96	30	19.69809	−27.5668
98	20	19.13559	−28.2404
98	25	18.77812	−27.9119
98	30	18.18377	−27.5708

**Table 6:** Average Von-Mises thermal stress changes with melt temperature.

V/P Switching Point	Melt Temperature/°C	Light Transmittance/%	Average Von-Mises Thermal Stress/MPa
94	20	92.825	28.51
94	25	95.508	27.39
94	30	96.300	26.28
96	20	92.221	28.71
96	25	94.203	27.57
96	30	96.187	26.42
98	20	92.420	28.64
98	25	93.042	27.50
98	30	96.345	26.37

**Table 7:** Relationship between volume contraction and fingerprint.

V/P Switching Point	Melt Temperature/℃	Average Volume Shrinkage/%	SD
94	20	6.232	1.194
94	25	6.328	1.147
94	30	6.424	1.100
96	20	6.312	1.202
96	25	6.403	1.154
96	30	6.496	1.106
98	20	3.373	1.198
98	25	3.157	1.151
98	30	2.895	1.103

## Data Availability

Not applicable.
